# Case Report: A patient harboring rare EGFR S768I/V769L compound mutation benefited from afatinib and osimertinib

**DOI:** 10.3389/fphar.2026.1714221

**Published:** 2026-01-23

**Authors:** Qingli Cui, Jiuzhou Zhao, Yichen Ma, Yanhui Hu, Dongyang Ma, Huaimin Liu

**Affiliations:** 1 Department of Integrated Traditional Chinese and Western medicine, Affiliated Cancer Hospital of Zhengzhou University & Henan Cancer Hospital, Zhengzhou, Henan, China; 2 Department of Molecular Pathology, Affiliated Cancer Hospital of Zhengzhou University & Henan Cancer Hospital, Zhengzhou, Henan, China; 3 The First Clinical Medical College of Xinjiang Medical University, Urumqi, Xinjiang, China

**Keywords:** afatinib, osimertinib, rare EGFR mutation, S768I and V769L, treatment

## Abstract

**Background:**

The development of epidermal growth factor receptor (EGFR) tyrosine kinase inhibitors (TKIs) dramatically altered the treatment for non-small cell lung cancer (NSCLC). The implementation of comprehensive genomic profiling for NSCLC facilitates to identify more uncommon genetic alterations in EGFR. S768I and V769L are two rare mutations in exon 20 of EGFR. However, the clinical reactivity of afatinib to the S768I/V769L compound mutation remains controversial, and there are no reports on whether osimertinib is effective against S768I/V769L. This case study aims to describe a clinical experience with these mutations, detailing the therapeutic strategy adopted and its outcomes, alongside a literature review to understand the broader implications for treatment.

**Case presentation:**

A 47-year-old man was referred to the Affilated Cancer Hospital of Zhengzhou University due to dry cough for more than 2 months and was diagnosed with Stage IIIB (T1cN3M0) lung adenocarcinoma. Mutation analysis of 26 lung cancer-related genes was performed using pathological tissue sample by targeted next-generation sequencing. A compound mutation of S768I and V769L in epidermal growth factor receptor exon 20 were observed. After multi-disciplinary treatment, the patient received concurrent chemoradiotherapy with pemetrexed and cisplatin, and achieved partial response. This patient did not receive durvalumab immunoconsolidation therapy for economic reasons. He developed disease progression 6 months after the end of radiotherapy. The patient was treated with afatinib at 40 mg daily by oral administration from January 2023. At the 1-month response assessment, the primary tumor in the right lung shrank and remained stable for 13 months. Magnetic resonance imaging revealed multiple nodules with brain metastases. We performed NGS testing of peripheral blood, and no mutation was found. Considering the superior intracranial efficacy of osimertinib, we tried high dose osimertinib for the patient. He achieved partial response after 15 days, and there were no intolerable adverse reactions. Three months later, the intracranial metastasis progressed, and headache appeared. The patient was switched to whole brain radiotherapy. The intracranial metastases remained stable after radiotherapy. The patient died 3 months later due to the progression of intracranial metastasis. overall survival was 26 months, slightly poorer than anticipated for patients with single driver gene mutations.

**Conclusion:**

The S768I/V769L mutation should be considered a poor-prognosis compound mutation. Patients with EGFR S768I/V769L compound mutated NSCLC may benefit from afatinib and osimertinib. Drugs with strong brain penetration capabilities are still needed for patients with S768I/V769L compound mutation to further improve survival outcomes.

## Introduction

The discovery of mutations in the epidermal growth factor receptor (EGFR) altered the clinical prognosis of non-small cell lung cancer (NSCLC). The common sensitizing exon 19 deletion (del19) and L858R EGFR mutations account for the majority of EGFR mutant NSCLC diagnoses. As next-generation sequencing (NGS) has become more increasingly utilized in the diagnoses of lung cancers, these uncommon EGFR mutations are being increasingly identified. 7%–23% of NSCLC harbor uncommon EGFR mutations (Keam et al., 2014; Kris et al., 2014; Yang et al., 2015). These mutations represent a highly heterogeneous group with 600 variants identified (Kobayashi and Mitsudomi, 2016). Up to 25% of uncommon EGFR mutation tumors coexist with other EGFR mutations within the same tumor and associated with poor clinical outcome in NSCLC (Kim et al., 2016).

Multiple phase III trials have demonstrated the superiority of EGFR-TKIs to chemotherapy for patients harbouring a common sensitizing *EGFR*-mutation (Mok et al., 2009; Sequist et al., 2013; Wu et al., 2017; Ramalingam et al., 2020). These landmark studies established EGFR-TKIs as first-line standard of care only included participants harbouring del19 and L858R EGFR mutations. However, there are limited prospective data on the efficacy of these drugs in patients with rare *EGFR*-mutations.

S768I and V769L are two rare mutations in exon 20 of EGFR. The S768I mutation is a point mutation causing serine (Ser) at position 768 in the EGFR protein kinase domain to be replaced by isoleucine (Ile). This position is adjacent to the C-helix (αC-helix) of the ATP-binding pocket, a structure crucial for maintaining the kinase’s activated conformation. Compared to other activating mutations, the kinase activation induced by S768I may be weaker or exhibit atypical characteristics. V769L is comparatively rare, resulting in the substitution of valine (V) at position 769 with leucine (L). The V769L mutation is adjacent to S768I and frequently co-occurs with it as a compound mutation, further diminishing the binding affinity of TKIs. In 2021, MD Anderson Cancer Center innovatively classified EGFR mutations into four distinct subgroups on the basis of sensitivity and structural changes: classical-like mutation, T790M-like mutation, exon 20 insertion mutation, and P-loop and αC-helix compressing (PACC) mutation. S768I and V769L were classified as PACC mutations (Robichaux et al., 2021).

The clinical reactivity of EGFR-TKIs to S768I/V769L compound mutation remains controversial. The initial report showed S768I/V769L have non responsiveness to gefitinib (Hajime et al., 2006). Two reports on afatinib (a second-generation EGFR-TKI) treatment of S768I/V769L have opposite effects, afatinib 20 mg proved ineffective, whereas afatinib 40 mg proved effective (Hao, 2019; Julie and Bruno, 2018). Although the NCCN guidelines have been approved the application of afatinib and osimertinib (a third-generation EGFR-TKI), for EGFR S768I mutation, there are limited clinical data for the efficacy of EGFR-TKIs in EGFR V769L. Up to now, there is no report on subsequent treatment in S768I/V769L. Herein, we present the first case report demonstrating that afatinib and osimertinib administrated sequentially can be effective long-term in this setting.

## Case report

**FIGURE 1:**
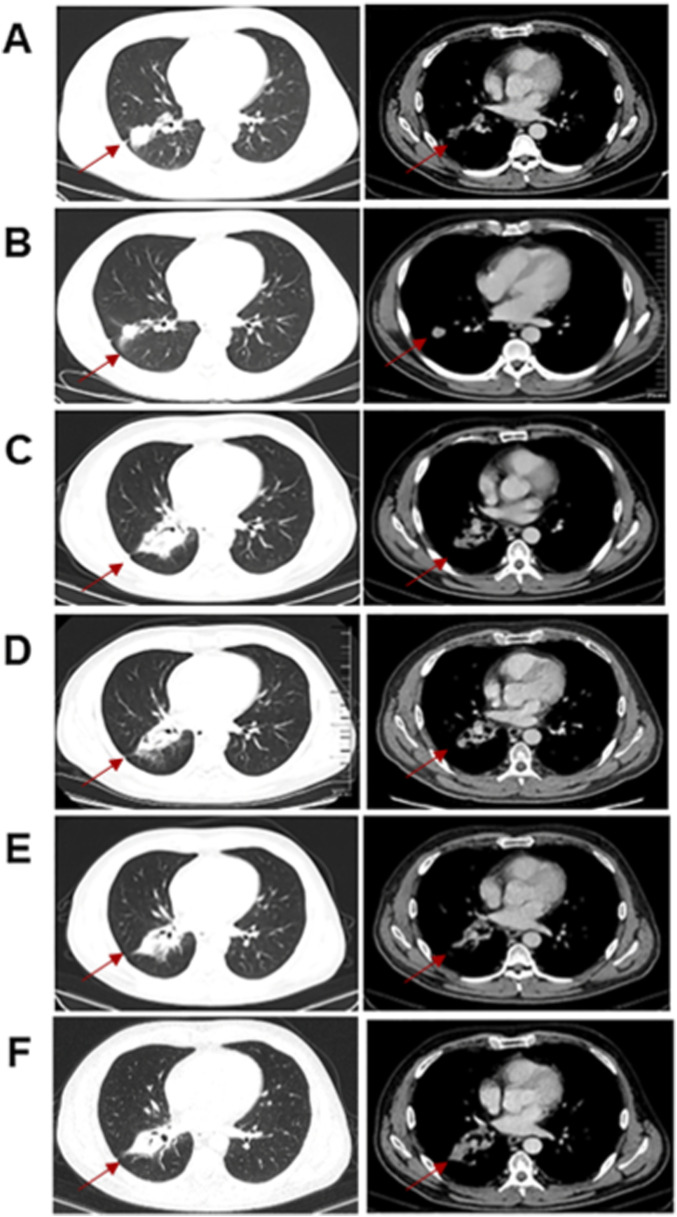
**(A)** Computed tomography (CT) scan of thorax before initiation of concurrent chemoradiotherapy. **(B)** CT scan of thorax after chemoradiotherapy. **(C)** CT scan of thorax before afatinib therapy. **(D)** CT scan of thorax after 1 month of afatinib therapy. **(E)** CT scan of thorax before osimertinib. **(F)** CT scan of thorax after 2 months of osimertinib.

**FIGURE 2:**
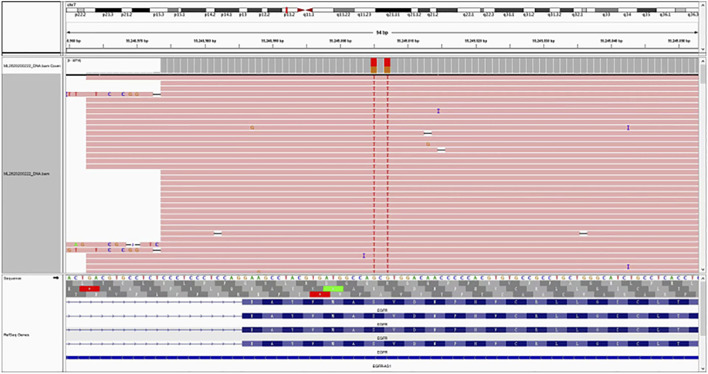
Next-generation sequencing analysis of S768I/V769L in EGFR exon 20 mutation in the patient’s aspiration biopsy sample.

A 47-year-old male patient with a 25-pack-year smoking history was admitted to our hospital in May 2022 after dry cough for more than 2 months. The patient has no family history of lung cancer. Computed tomography (CT) revealed a malignant mass in the right lobe with mediastinal, hilar and right supraclavicular lymphadenopathy (Figure 1A). No distant metastases were found. Lung biopsy and right supraclavicular lymph node biopsy support the diagnosis of lung adenocarcinoma. Mutation analysis of 26 lung cancer-related genes was performed using pathological tissue sample by targeted NGS. A compound mutation of S768I and V769L in EGFR exon 20 were observed (Figure 2), and variant allele frequency was 55.17%. Both the 22C3 and SP263 kits indicated a tumor proportion score (TPS) of <1% for PD-L1 detection.

**FIGURE 3:**
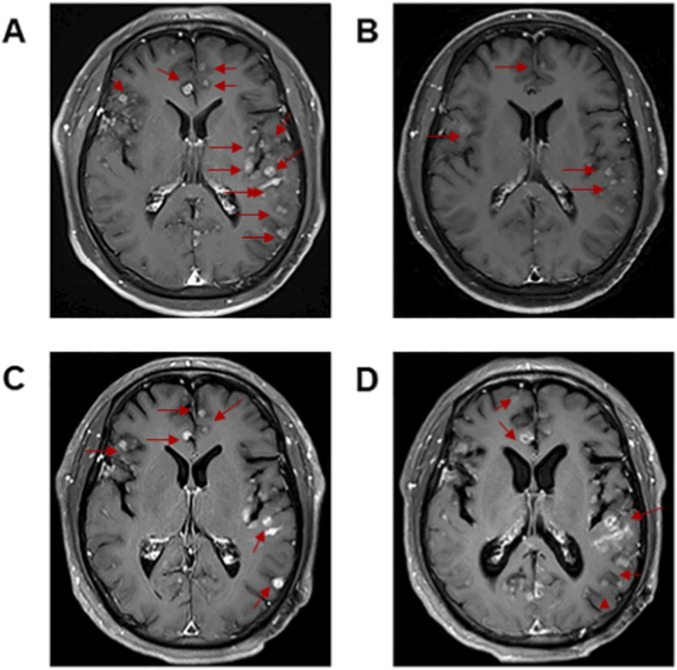
**(A)** Magnetic resonance imaging (MRI) scan of the brain before osimertinib therapy. **(B)** MRI scan of the brain after 15 days of osimertinib therapy. **(C)** MRI scan of the brain after 3.5 months of osimertinib therapy. **(D)** MRI scan of the brain after whole brain radiotherapy.

Stage IIIB (T1cN3M0) NSCLC was diagnosed in the patient. After multi-disciplinary treatment, the patient therefore received concurrent chemoradiotherapy with pemetrexed and cisplatin, and achieved partial response (Figure 1B). However, half a year after chemoradiotherapy ended, chest and abdominal CT showing enlarged primary tumor lesions (Figure 1C). The patient was treated with afatinib at 40 mg daily by oral administration from January 2023. He suffered moderate rash and mild diarrhea to afatinib. At the 1-month response assessment, the primary tumor in the right lung shrank and remained stable (Figure 1D). Follow-up examinations were conducted every 3 months, comprising chest and abdominal CT scans (Figure 1E) and cranial MRI scans. Brain metastases were detected during the scheduled follow-up in March 2024. Magnetic resonance imaging (MRI) revealed multiple nodules with brain metastases (Figure 3A). No symptoms of brain metastases were present at that time. Because the patient refused to have a second biopsy, we did not perform tissue genetic testing. We performed NGS testing of peripheral blood, and no mutation was found. Beyond switching to a more brain-penetrant targeted therapy or administering local radiotherapy, there are few better options available to halt the progression of brain metastases. Based on the results of a phase 2 clinical study (Zofia et al., 2020), the patient was then treated with osimertinib at 160 mg daily from March 2024. He achieved partial response after 15 days (Figure 3B), and there were no intolerable adverse reactions.

After two months of treatment with osimertinib, the lung lesions remained stable (Figure 1F). Unfortunately, 3 months later, the intracranial metastasis progressed (Figure 3C), and headache appeared. The patient was switched to whole brain radiotherapy. The intracranial metastases remained stable after radiotherapy (Figure 3D). The patient died 3 months later due to the progression of intracranial metastasis.

## Discussion

NSCLC constitutes approximately 80% of new lung cancer cases, and approximately one-third of these patients have stage III (locally advanced) disease at the time of their diagnosis. Five-year overall survival (OS) for patients with unresectable stage III NSCLC is poor. The management of unresectable stage III NSCLC has evolved dramatically over the past 2 decades. Until recently, a standard of care for patients in this setting was treatment with concurrent platinum-based chemotherapy and radiation (Auperin et al., 2010). Unfortunately, despite this treatment, OS in this population is poor with a survival rate of only approximately 15% at 5 years (Auperin et al., 2010; Ahn et al., 2015). Prior to the publication of the PACIFIC trial, the efficacy of standard chemoradiation in unresectable stage III NSCLC had reached a plateau, with studies evaluating radiation dose-escalation and consolidative chemotherapy strategies failing to improve outcomes (Bradley et al., 2015; Hanna et al., 2008; Ahn et al., 2015). The PACIFIC trial found consolidative durvalumab after chemoradiation to significantly improve progression-free survivial (PFS) and OS, and now represents a current standard of care for the treatment of unresectable stage III NSCLC (Antonia et al., 2017; Antonia et al., 2018; Spigel et al., 2022).

The EGFR is a critical target in NSCLC treatment. While stage III NSCLC with common EGFR mutations such as del19 and L858R mutations are well-known and have established treatment protocols, rare mutations pose significant therapeutic challenges. The LAURA study confirmed third-generation EGFR-TKI-targeted consolidation therapy after radical chemoradiotherapy as the new standard of care for patients with stage III unresectable EGFR-mutated (del19/21L858R) NSCLC (Lu et al., 2024). However, there is limited data pertaining to the safety and efficacy of this approach in patients with EGFR rare mutations.

The increasing use of NGS techniques are likely to expand the detection of targetable somatic mutations throughout EGFR. Moreover, the implementation of plasma-based mutation detection assays will facilitate increased uptake of baseline EGFR mutation testing. The S768I point mutation accounts for 1.5%–3% of untreated EGFR-mutated tumors (Kobayashi and Mitsudomi, 2016). It is a major uncommon mutation confers sensitivity to afatinib. EGFR V769L is generally considered to be a rare mutation resistant to EGFR-TKIs (Kobayashi and Mitsudomi, 2016). Prior case reports of *de novo* EGFR S768I and V769L compound mutations have been published with mixed responses to first- and second-generation EGFR TKIs.

Julie and Bruno (2018) reported a patient with S768I/V769L resistant to afatinib and inferred that V769L mutation induces resistance to second-generation EGFR TKIs. Hao (2019) reported a patient with S768I/V769L mutation who response to full dose of afatinib and PFS was more than 11 months. They also conducted virtual molecular docking and inferred that the S768I/V769L mutation had little impact on the affinity of afatinib.

This patient was diagnosed with stage III unresectable NSCLC with S768I/V769L mutation while the LAURA study was still recruiting, and treatment modalities patients with EGFR mutations are still being explored. Referring to the PACIFIC treatment model recommended by the guidelines, this patient underwent concurrent chemoradiotherapy, did not receive durvalumab immunoconsolidation therapy for economic reasons. He developed disease progression 6 months after the end of radiotherapy.

The varied incidence of uncommon *EGFR* mutant advanced NSCLC has meant that there is limited prospective clinical trial data that evaluates the efficacy of EGFR TKIs in patients harbouring uncommon *EGFR* mutations. Preclinical data has shown that osimertinib is active against most common EGFR mutations but the IC50 values of osimertinib against uncommon EGFR mutations, including the S768I mutation, were higher than those of afatinib *in vitro* study (Banno et al., 2016). The combined *post hoc* analysis of LUX-Lung 2, LUX-Lung 3, and LUX-Lung 6 trials demonstrated that NSCLC patients harbouring S768I mutation treated with afatinib had an objective response rate (ORR) of 100%, with a median PFS of 14.7 months (Yang et al., 2015). Both a retrospective and prospective phase 2 non-randomised study (UNICORN) demonstrated clinical activity of osimertinib in uncommon *EGFR* mutations with an ORR of up to 66% (Bar et al., 2023; Okuma et al., 2024). A *post hoc* subgroup analysis of pooled data from two phase II clinical trials found Osimertinib was active in patients with uncommon EGFR mutations, and especially for G719X-compound mutations (Eide et al., 2022). Despite this, none of the trials included patients with S768I/V769L compound mutation.

This patient received full dose afatinib for 13 months. However, options for subsequent targeted therapy after progression on afatinib in such patients are limited, particularly in patients without the T790M mutation. For our patient, treatment options are very limited when intracranial progressed. Second-generation TKIs have limited activity in the central nervous system, demonstrating the need for novel TKIs with CNS activity.

There are no reports on whether osimertinib is effective against S768I/V769L. Schoenfeld et al. (2020) identified an array of acquired resistance mechanisms to osimertinib using paired pre- and post-treatment tissue samples. They identified the EGFR S768I/V769L compound mutation as one of the resisitance mechanisms to osimertinib. It is unclear if treating a patient who has naïve EGFR S768I/V769L compound mutation will respond to osimertinib.

Considering the superior intracranial efficacy of osimertinib, we tried high dose osimertinib in this occurrence. Our patient benefited from high dose osimertinib therapy for 3.5 months. Despite this patient received whole brain radiotherapy after progression on osimertinib. Unfortunately, this patient ultimately died from intracranial progression. OS was 26 months, slightly poorer than anticipated for patients with single driver gene mutations.

This report has several limitations. Tissue was prioritized for genetic profiling after disease progression. We used peripheral blood samples to assess ctDNA using NGS because the patient refused tissue biopsy. The difference in the panel size of the genes in two tests can influence the depth of sequencing coverage and the possibility of further exploring mechanisms of resistance. In addition, whether leptomeningeal metastasis occurs during disease progression remains uncertain. Osimertinib had a greater ability to penetrate the blood-brain barrier and was more effective against brain metastases and meningeal carcinomatosis in patients with EGFR mutation-positive NSCLC than first- or second-generation EGFR TKIs. We suspect that the relatively short PFS from osimertinib and radiotherapy in this patient maybe associated with the presence of leptomeningeal metastasis. For this patient, it is difficult to determine whether immunotherapy consolidation following radiotherapy and chemotherapy would improve overall survival, whether intrathecal chemotherapy would improve overall survival, or whether combining bevacizumab upon detection of brain metastases would yield better intracranial control.

To the best of our knowledge, this is the first case with S768I/V769L mutation has benefited from sequential treatment with afatinib and osimertinib. Our case highlights a potentially effective strategy after the development of afatinib resistance among patients harboring S768I/V769L mutation. No T790M was detected in this patient, largely excluding the possibility that the response to osimertinib was due to its ability to specifically target T790M. We therefore inferred from these results that S768I/V769L is possibly an activating mutation to afatinib and osimertinib. It would be interesting to explore high-dose of osimertinib in first-line treatment of NSCLC harboring S768I/V769L mutation.

## Conclusion

Our observation suggests that patients with EGFR S768I/V769L compound mutated NSCLC may benefit from afatinib and osimertinib. We believe this manuscript is valuable for all the researchers who are interested in rare mutation of NSCLC.

## Data Availability

The raw data supporting the conclusions of this article will be made available by the authors, without undue reservation.
